# SARS-CoV-2 Transmissibility Within Day Care Centers—Study Protocol of a Prospective Analysis of Outbreaks in Germany

**DOI:** 10.3389/fpubh.2021.773850

**Published:** 2021-12-13

**Authors:** Anja Schienkiewitz, Susanne Jordan, Anselm Hornbacher, Hanna Perlitz, Marie-Luise Zeisler, Anna Sandoni, Ulrike Kubisch, Barbara Wess, Tim Kuttig, Angelika Schaffrath-Rosario, Stefan Damerow, Petra Rattay, Gianni Varnaccia, Anne-Kathrin M. Loer, Jan Wormsbächer, Carolin Cohrdes, Matthias Wetzstein, Stefan Albrecht, Isabell Hey, Janine Michel, Livia Schrick, Antje Gößwald, Jennifer Allen, Martin Schlaud, Markus A. Busch, Hans Butschalowsky, Jörg Wernitz, Eveline Otte im Kampe, Udo Buchholz, Walter Haas, Lars Schaade, Lothar H. Wieler, Thomas Ziese, Thomas Lampert, Julika Loss

**Affiliations:** ^1^Department of Epidemiology and Health Monitoring, Robert Koch Institute, Berlin, Germany; ^2^Centre for Biological Threats and Special Pathogens, Robert Koch Institute, Berlin, Germany; ^3^Department of Infectious Disease Epidemiology, Robert Koch Institute, Berlin, Germany; ^4^Leadership Robert Koch Institute, Robert Koch Institute, Berlin, Germany; ^5^Department of Methodology and Research Infrastructure, Robert Koch Institute, Berlin, Germany

**Keywords:** children, day care center, SARS-CoV-2, COVID-19, symptoms, susceptibility, transmission, secondary infection rate

## Abstract

**Introduction:** Until today, the role of children in the transmission dynamics of SARS-CoV-2 and the development of the COVID-19 pandemic seems to be dynamic and is not finally resolved. The primary aim of this study is to investigate the transmission dynamics of SARS-CoV-2 in child day care centers and connected households as well as transmission-related indicators and clinical symptoms among children and adults.

**Methods and Analysis:** COALA (“Corona outbreak-related examinations in day care centers”) is a day care center- and household-based study with a case-ascertained study design. Based on day care centers with at least one reported case of SARS-CoV-2, we include one- to six-year-old children and staff of the affected group in the day care center as well as their respective households. We visit each child's and adult's household. During the home visit we take from each household member a combined mouth and nose swab as well as a saliva sample for analysis of SARS-CoV-2-RNA by real-time reverse transcription polymerase chain reaction (real-time RT-PCR) and a capillary blood sample for a retrospective assessment of an earlier SARS-CoV-2 infection. Furthermore, information on health status, socio-demographics and COVID-19 protective measures are collected via a short telephone interview in the subsequent days. In the following 12 days, household members (or parents for their children) self-collect the same respiratory samples as described above every 3 days and a stool sample for children once. COVID-19 symptoms are documented daily in a symptom diary. Approximately 35 days after testing the index case, every participant who tested positive for SARS-CoV-2 during the study is re-visited at home for another capillary blood sample and a standardized interview. The analysis includes secondary attack rates, by age of primary case, both in the day care center and in households, as well as viral shedding dynamics, including the beginning of shedding relative to symptom onset and viral clearance.

**Discussion:** The results contribute to a better understanding of the epidemiological and virological transmission-related indicators of SARS-CoV-2 among young children, as compared to adults and the interplay between day care and households.

## Introduction

### Background

At the beginning of the COVID-19 pandemic in spring 2020, children under 6 years accounted for only a small proportion of laboratory-confirmed COVID-19 cases (1–4). In many countries, day care centers were closed early on, so it was unclear whether the low incidence among children could be explained by the lack of contacts among young children outside the household. As children are also prone to show milder symptoms in comparison to adults, infections might have remained unrecognized (5). Yet it is well established that children infected with SARS-CoV-2 can transmit the virus (6). Published data about the role of children and exposure settings on the transmission dynamics of SARS-CoV-2 implies that adults may exert a stronger influence on the transmission dynamics than children (6–10).

In Germany, 35% of the children under the age of 3 years and 92.8% of 3–6-year-old children attended center-based care in the year 2020 (about 3.4 million children) (11, 12). Day care centers are usually set in an indoor environment with sustained close contacts among children and staff, potentially creating a high-risk setting for infections with SARS-CoV-2. A better understanding of the factors fostering SARS-CoV-2 transmission in and from day care centers is key to developing tailored measures to control the pandemic and to avoid closures of day care centers or day care groups.

Closures of day care centers can have adverse consequences on small children's educational and pedagogical needs as well as on their social and mental well-being (13, 14). In order to be able to respond to infections in a flexible manner and to, at that time, assumably reduce the number of secondary infections within day care centers, the structuring of day care into separated groups became mandated to reduce the risk of transmission and the rapid spread of the virus. Through the restructuring of day care into small and isolated groups, the number of exposed people to a possible primary case could be reduced and infection chains became more manageable. In that consequence, quarantine measures could be applied more specifically and therefore affect a smaller number of people.

The transmission dynamics among children and infections occurring from children to adults are still poorly understood and emerging variants of SARS-CoV-2 do indicate a potential for enhanced infectivity in younger age groups (15, 16). This research gap concerns the situation in day care centers regarding children and the adult day care center staff. A better understanding of the factors fostering SARS-CoV-2 transmission in and from day care centers is necessary for the development of more pronounced and tailored measures against the virus.

### Aims and Objectives

In order to gain a better understanding about the susceptibility (receptivity) and infectiousness of children and adults in day care settings, biological, behavioral, and contextual factors of the source case and close contacts as well as the varying factors of different exposure conditions are investigated. The study addresses the following questions:

1. How infectious are children of 1 to 6 years tested positive for SARS-CoV-2 with regard to close contacts in day care and household (children and adults)? How susceptible are children of 1 to 6 years to SARS-CoV-2 infections among close contacts in the day care center?
  a. In cases of SARS-CoV-2 events in day care settings, what is the rate of secondary transmissions among exposed children and adults in the respective day care center group and households?
  b. What is the duration of viral shedding (as measured by real-time RT-PCR) among infected children 1 to 6 years of age, as compared to infected adults from the same settings?
  c. What are sero-conversion rates among children 1 to 6 years old with PCR-confirmed SARS-CoV-2 infections, as compared to infected adults (e.g., day care center staff or adult members of affected households)?
2. What are the symptoms of infected 1 to 6 year old children, as compared to adults? What is the proportion of symptomatic and asymptomatic COVID-19 infections among children and adults?
3. How important is the baseline health status (e.g., presence of chronic diseases) for the degree of severity of SARS-CoV-2 infections among children and adults?
4. Do the included day care centers vary in care concepts and hygiene measures?

## Methods and Analysis

The “Corona outbreak-related examinations in day care centers—COALA” is a project within the interdisciplinary “Corona Day Care Study,” which is conducted by a collaborative research consortium of the German Youth Institute (DJI) and the Robert Koch Institute (RKI).

### Study Design and Case Definition

**Figure 1:**
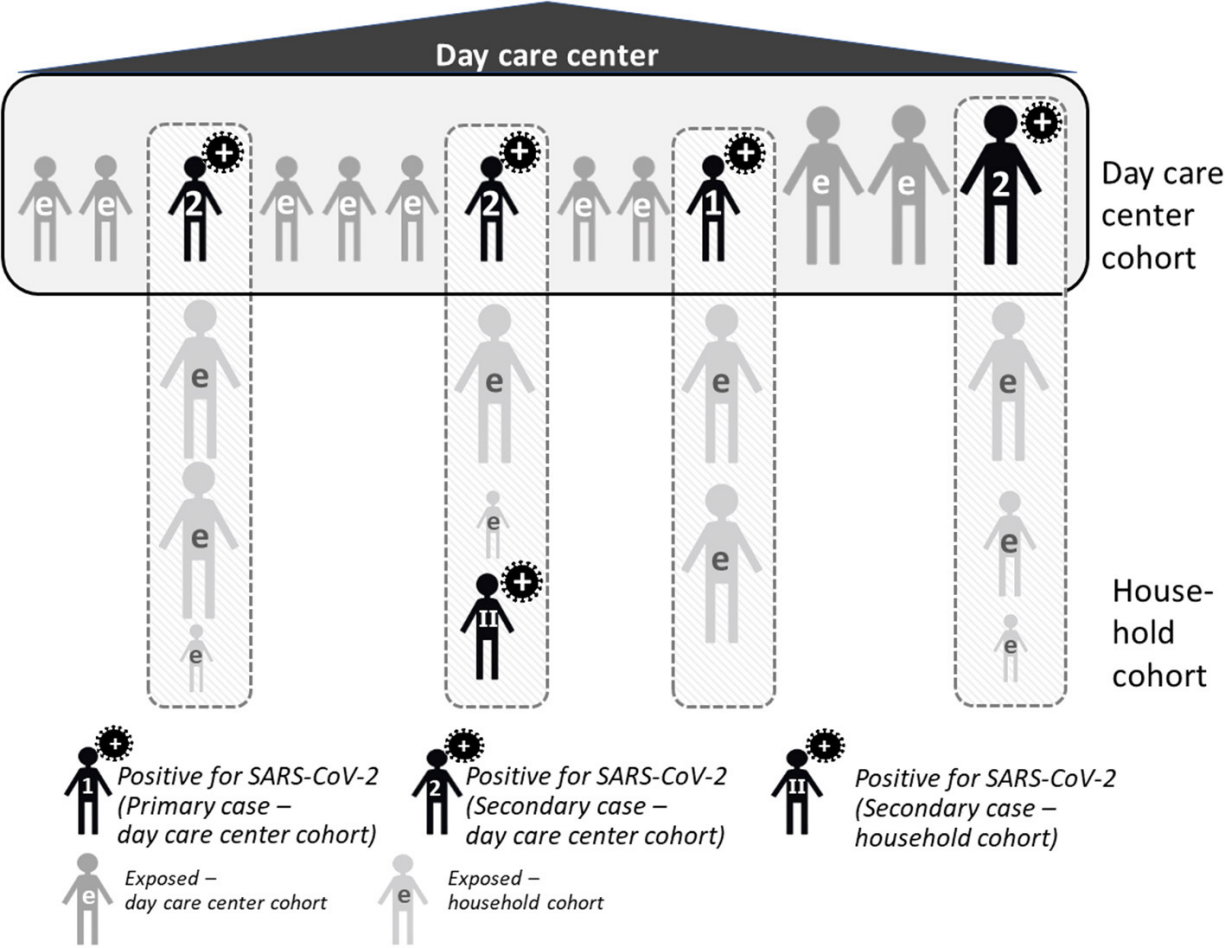
Study design of COALA. e, exposed.

COALA uses a case-ascertained study design in day care center groups and households, i.e., a primary case has been diagnosed (“ascertained”) and is observed together with exposed persons in the household and in the day care center group over a period of time—in our case, 12 days. In this period, mouth and nose swabs are taken, and symptoms documented, which is done initially by members of the study team, and in the course of the following 12 days by the participants and/or their legal guardians themselves. Case-ascertained studies are well-suited for assessing how the virus spreads as comparatively small sample sizes are sufficient for the observation of secondary transmissions (17, 18). COALA addresses two populations within the case-related study design: Day care centers (so-called day care center cohort) and associated households (so-called household cohort, see Figure 1).

In the COALA study, the primary case is defined as the first SARS-CoV-2 positive case in the day care center group. It can be either a child between 1 to 6 years or an adult (staff member) from the day care center. The index case is defined as the first case within the day care group that is diagnosed with SARS-CoV-2 and reported to the local health authority. Primary and index case can be identical; however, it is possible that the primary case remains undetected (e.g., because of lack of symptoms), and only a secondary case with symptoms gets tested and is diagnosed first. On the basis of the information provided by the local health authority, the director or pedagogic staff of the day care center management and the study participants, the respective primary case is identified for each day care center. In addition, each infected person in the day care center group (primary or secondary case, child or staff member) may potentially spread the infection to his or her respective household members. On the level of the household, this SARS-CoV-2 case is the primary case for the respective household members.

### Level of Day Care Centers (Day Care Center Cohort)

In case of a verified and officially reported SARS-CoV-2 case within a day care center, close contacts to the primary case and the primary case themselves are included into the study (so-called day care center cohort). Normally, all children and staff members who belong to a group within the day care center can be defined as “close contacts”, as they spend many hours in a room together every day, playing with each other and the implementation of preventive measures within the group is limited. To identify (further) subsequent cases (at an early stage), close contacts of the primary case of the day care center and their family members are observed over a period of 12 days, including monitoring of viral load by regular swabs, and the development of symptoms relevant to COVID-19, which are documented in daily symptom diary.

### Level of Households (Household Cohort)

Children and staff of the day care center who test positive for SARS-CoV-2 are included in the household cohort, as are all members of their household, who are regarded as a close contact to this primary household case infected within the day care center. These positive cases from the day care center are defined as the primary case in their respective household, unless the laboratory findings, a positive result at initial testing, a history of exposition and/or sequence of clinical symptoms may point to another positive case in the household who has been infected earlier. As in the day care center cohort, the persons infected with SARS-CoV-2 as well as their household contacts regularly take swabs and document their symptoms over 12 days. This procedure helps to track the viral load and to record the longitudinal development of symptoms over time of those infected, and can identify secondary cases early.

Children or staff members with contact to a positive case but initially tested negative for SARS-CoV-2 are included in the study, but not the members of their households. In case one of participants diagnosed with a SARS-CoV-2 infection during the study period (as a secondary case), their household members are also included in the study as part of the household cohort, being close contacts to this household case.

### Inclusion/Exclusion Criteria

Inclusion criteria for a day care center into the COALA study are

1. A case of SARS-CoV-2 within a day care center (=index case) is reported to the responsible local health authority.
2. The research team is informed about the case no longer than 3 days after the diagnosis, so that local testing of close contacts can start no later than 4 days after identification of this index case and the maximum of 5 days after the PCR test is not exceeded.

Inclusion criteria for individuals are

- minimum age of 1 year
- being either the index case in the day care center, or being a child or a staff member of the day care center group of the index case (= close contact, due to the nature of interactions within a day care center group)
- being a household member of a SARS-CoV-2 positive person (both primary and secondary cases), i.e., partners, siblings and other persons who stay a minimum of 4 nights per week in this household

Exclusion criteria are

- age younger than 1 year
- insufficient knowledge of the German language
- substantial health problems that do not allow any collection of specimens (e.g., severe disease, injury)

### Recruitment

The study team is informed about the positive cases directly by cooperating local health authorities, or by the day care centers themselves. In the latter case, the study team contacts the local health authority in charge to verify the case and receive more details related to the case or outbreak. If parents and staff members consented to being contacted by the study team, they are invited to participate in COALA, receive oral information (by phone) and written material explaining the study (via e-mail). Different information documents are available, tailored to the literacy levels and preferences of different age groups (1–5, 6–11, 12–17 years, adult). The information material for ages 6 to 17 years may be used for older siblings in the households. If the contacted parents or staff members agree to participate, a home visit of members of the study team is scheduled. The parents or staff members are either in quarantine respectively in isolation themselves (as cases or close contacts of a case), or their small child is in quarantine, and needs to be cared for at home. Therefore, a home visit is an appropriate way to make sure to meet, and test, all relevant household members.

COALA includes day care centers from all over Germany.

### Procedure of Collecting Data and Biological Samples

Data and biological specimens are collected from the study participants in different steps:

1. an initial home visit by the study team
2. a telephone-based interview
3. a period of self-sampling and symptom logging by the study participants
4. a second home visit by the study team (only for participants tested positive for SARS-CoV-2).

For the initial examination, all participants are visited at home by a trained study team. The first home visit takes place on day 4, 5 or 6 (=t1) after testing date of the index case (t0). These home visits include (1) study information, informed consent, (2) collection of a combined mouth and nose swab, a saliva sample and capillary blood sample which can be used for a retrospective assessment of an earlier SARS-CoV-2 infection (19). This blood sample is taken from the fingertip and dried on specific paper for transport (dried blood spot). Furthermore, the members of the study team (3) distribute handouts and explanations on how to complete the symptom diary for the following 12 days after the home visit, (4) instruct household members in self-sampling and sampling children (mouth and nose swab, saliva sample). The self-sampling is performed four times, every third day (t2, t3, t4, t5), and the biological samples and the symptom diary are returned to the study team by mail. 0–3 days after the home visit (t1), telephone interviews are conducted to collect data about all study participants on socio-demographics, health status and protective behavior during the pandemic (see section Standardized Interviews). At t2, a stool sample of children is taken to allow for the potential identification of prolonged viral shedding in cases of infection when respiratory samples may already be negative after few days, which may happen in children (20, 21).

**Figure 2:**
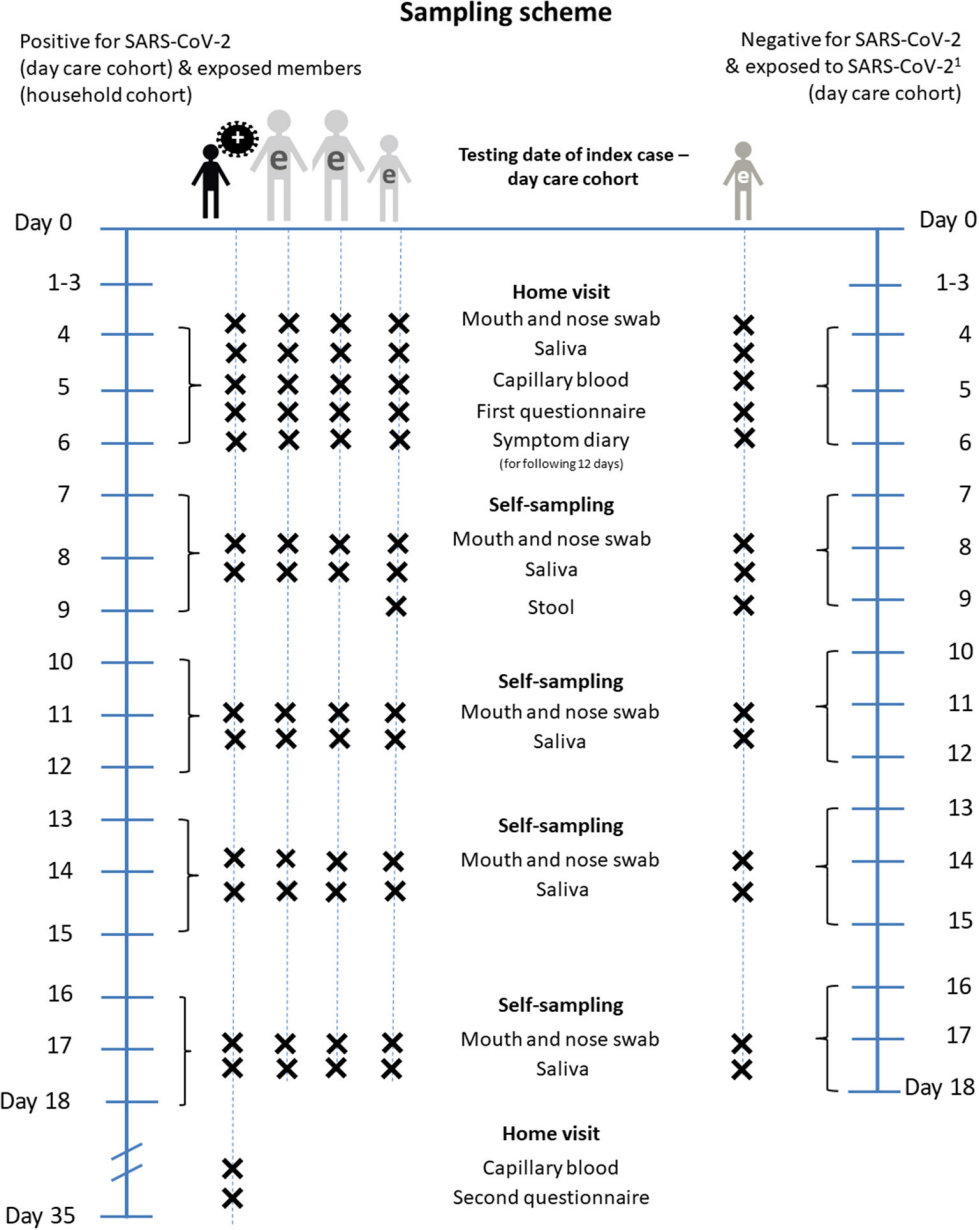
Sampling scheme of COALA. Day 0, testing date of the index case (=Baseline). e, exposed person. ^1^For exposed children and day care personnel newly positive for SARS-CoV-2 until day 13 to 15, the procedure is the same as for initially positive day care personnel and households from day 7 to 9.

Participants are either individuals tested positive for SARS-CoV-2 within the day care setting (children, adults/staff), or close contacts of those cases, both from the day care group (day care center cohort) and from the households of positive cases (household cohort, see Figure 2). The sampling scheme is identical for positive cases and close contacts. The only difference is an additional stool sample in children tested negative for SARS-CoV-2 at t2 to detect missed SARS-CoV-2 infections among children.

Approximately 35 days after the test of the index case, every study participant with a positive PCR test result for SARS-CoV-2 during the course of the COALA study or previously confirmed through local health authorities is re-visited at home. A capillary blood sample from the fingertip is taken for a final test on sero-conversion of SARS-CoV-2 antibodies. A standardized interview is conducted for a retrospective assessment of the clinical course of the disease (e.g., on COVID-19-related hospital stays).

### Detection of New SARS-CoV-2 Infections in the Study

The participants are repeatedly tested for SARS-CoV-2, which may lead to the detection of new cases among close contacts. Due to the constantly changing pandemic situation, local health authorities cannot always guarantee SARS-CoV-2 testing of all exposed persons, but need to limit their contact tracing to informing close contacts and sending them into quarantine. In this case, many study participants may have an unknown infection status when recruited. The PCR tests during the home visit (t1) may then identify additional SARS-CoV-2 cases among the participants. New cases may also be detected later on in the course of the study, e.g., a secondary transmission in the household cohort. In any of these cases, the infected person as well as the local health authority will immediately be notified by phone, and the authority will need to put the person in isolation or quarantine, respectively. If the newly diagnosed infection represents a primary case in the household cohort (e.g., a child of the day care center group which has been tested negative before), then all household members are considered close contacts of this case and are asked to undergo the self-test regime as described above. During the initial home visit, household members are prepared for this possible scenario and provided with the necessary test equipment. Primary cases in day care centers and households are identified on the basis of laboratory testing results and information provided through the different questionnaires. For symptomatic cases the likely date of infection is estimated by the onset of symptoms and the mean incubation period. For asymptomatic cases, we assume that the infection event has occurred up to 10 days before the first laboratory-confirmed SARS-CoV-2 test result.

### Biological Samples

In order to detect SARS-CoV-2 infections among participants, combined mouth and nose swabs, saliva samples and stool samples are tested for viral RNA by real-time RT-PCR. In order to assess sero-conversion, blood samples are tested for IgG antibodies against the S1 domain of the SARS-CoV-2 spike protein with a semiquantitative ELISA (see Table 1).

**Table 1:** Biological samples and laboratory examinations.

**Sampling**	**Endpoint**	**Primary measure**	**Aim**
Mouth and nose swab/saliva	Detection of SARS-CoV-2	Real-time RT-PCR-based detection of viral RNA	Detection of (secondary) transmission of SARS-CoV-2 to close contacts
Mouth and nose swab/saliva	Viral load	Ct-value	Estimating the time course of potential SARS-CoV-2 infectiousness among positive persons
Stool	Detection of SARS-CoV-2	Real-time RT-PCR-based detection of viral RNA	Detection of missed SARS-CoV-2 infections among children due to prolonged fecal viral shedding
Mouth and nose swab/saliva/stool	Genome typing of SARS-CoV-2 RNA	Virus variants of SARS-CoV-2	Spread of Variants of Concern (VOC), evaluation of the importance of variants for secondary transmission and infectiousness
Capillary blood	Detection of antibodies against SARS-CoV-2	ELISA-based, semiquantitative detection of IgG-antibodies against the S1 domain of the SARS-CoV-2 spike protein	Determination of susceptibility to SARS-CoV-2 infections among close contacts; detection of the SARS-CoV-2 sero-conversion

Mouth and nose swabs and saliva samples are used because (a) both procedures are less invasive than nasopharyngeal or oropharyngeal swabs and are therefore more suitable for very young children (mouth swaps are dipped into medical glucose solution), and (b) they are an easier and more reliable method for self-testing. The combination of mouth and nose swabs and saliva samples has been shown to be of similar quality to a deep nasopharyngeal swab (22–26). The initial swabs are taken by trained staff during the first home visit. Subsequent samples are taken by the participants themselves using the collection and transport equipment handed out during the first home visit.

Children who are tested negative at baseline and are classified as close contacts to the primary case of the day care center are asked to provide an additional stool sample at the first self-testing date, about 3 days after the first home visit. The sample is taken either from the stool catcher or from the diaper or potty in the case of very young children (Fa. Süsse Labortechnik: stool catcher for sampling; Fa. Sarstedt: stool tube 76 x 20 mm, sterile).

The blood sample is taken by trained study personnel in a quiet, non-intimidating environment, with children up to 6 years of age preferably sitting on the parent's lap. The blood sample is taken using a safety lancet (Unistik 3 Neonatal, safety lancet 18 G, 1.8 mm for infants and Unistik 3 EXTRA 21 G, 2.0 mm for children from 6 to 8 years of age and adults) onto the blood collection set for dried capillary blood samples manufactured by Euroimmun (27, 28).

All self-testing samples are sent to the RKI laboratories by mail in a lockable plastic bag inside a rigid cardboard box. The packaging and shipping material are provided to all households during the first home visit. The participants do not have to pay any postage costs.

### Documentation of COVID-19 Symptoms

After the initial measurements during the home visits, household members record clinical symptoms for a period of 12 days, documenting symptoms such as fever ≥ 38°C, chills, cough, shortness of breath, muscle aches, nausea, diarrhea, olfactory dysfunction, gustatory dysfunction as well as other complaints that may not have been listed.

### Infection Protection During Home Visits

During the home visits the participants are asked to wear non-medical, fabric masks or medical masks, which are provided to them if needed. Participants are also asked to maintain a physical distance and to provide ventilation during the visit. The study team members work in personal protective equipment (PPE). Extensive Standard Operating Procedures (SOP) and training documents are available for the occupational safety and infection protection measures.

### Risks and Benefits for Participants

Examinations have minimal risk for participants. The direct benefit is a regular feedback on SARS-CoV-2 infection status. All participants receive a voucher for the first home visit (50 Euros) and in case of a second home visit another voucher (25 Euros). In addition, all households with children receive a small non-monetary gift (e.g., toy). If there is a positive PCR test result of samples collected during the home visit or later on in the course of the study, the infected person as well as the local health authority will immediately be notified by phone within 24 h. Six weeks after first home visit all participants receive a result report with PCR test results for SARS-CoV-2 virus material and for SARS-CoV-2 IgG antibodies.

Participants and the public will benefit from the data collected by improving the understanding of SARS-CoV-2 transmission dynamics among children in day care centers and their corresponding households, as well as the clinical course of SARS-CoV-2 infection in children of day care age.

### Standardized Interviews

Information on the exact occurrence of the outbreak within the day care center as well as additional information on the participant's health status and the course of symptoms associated with SARS-CoV-2 are assessed with different instruments during the study. Local health authorities, the director or pedagogic staff of the day care center management and household members are contacted via telephone for a computer-assisted telephone interview (CATI). For children under the age of 14 years, parents or legal guardians, respectively, should answer the questionnaire on behalf of their children. The final interview—only for SARS-CoV-2 positive participants—is conducted through a paper or computer-assisted personal interview (PAPI/CAPI) during the second home visit. An overview of all questionnaires is given in Table 2.

**Table 2:** Overview of questionnaires in COALA.

**Questionnaire for**	**Day care center**	**Households**					**Local Health Authorities**
Sampling period		Baseline measurement				Final measurement	
Interview mode^*^	CATI	CATI				PAPI/CAPI	CATI
Point of time	**t1–t2**	**t1–t2**				**t6**	**t6**
Target person	Director	Representative of household (mostly parents, legal guardians)	Child under age 14 years	Additional household member aged 18+ years	Adolescent aged 14+ years	All participants tested positive for SARS-CoV-2	Technical officer disease control and prevention department
Person to be inter-viewed	Director or pedagogic staff	Child's parents/legal guardians or staff from day care center	Parents/legal guardians	Partner(s)/grandparents or any other adult member of the respective household	Siblings	Legal guardians/adult member of the household, children aged 14+ years	Technical officer who is familiar with the outbreak
Questionnaire main content	Structure of day care center, e.g., group size, number of groups and rooms, SARS-CoV-2 protective behavior	Household composition, health risk assessment, SARS-CoV-2 protective behavior; mental stress due to quarantine	Test results, health risk assessment	Test results, health risk assessment, mental stress due to quarantine	Test results, health risk assessment	Retrospective information of course of COVID-19 disease, reasons for missing data	Epidemiological assessment of the outbreak: testing, test results, index cases, primary cases

* *CATI, computer-assisted telephone interview; PAPI/CAPI, paper-assisted personal interview/computer-assisted personal interview*.

### Quality Management

Standard Operating Procedures (SOP) are prepared for all steps of the data collection process. A regular supervision of the data collection is assured by continuous supervision and training of the study teams. If required, additional individual trainings are conducted. All procedures regarding laboratory examinations from blood collection to the arrival of the participants biological samples are documented.

### Data Analysis

For answering questions about infectiousness of children and adults in day care centers and households, secondary attack rates (SAR) are calculated (which can also be named as secondary infection rates). The SAR is understood as the proportion of PCR-positive cases among all persons exposed to the primary case (29). SARs are stratified and evaluated according to age group (children, adults) and exposure setting (day care center, household). Furthermore, the SAR of asymptomatic primary cases is compared with the SAR of symptomatic primary cases. Serial intervals, defined as the time interval between the start of clinical symptoms in primary cases and the start of symptoms in secondary cases, are also estimated. The development of antibodies which can be determined with the antibody test after SARS-CoV-2 infection takes different periods of time and is known as sero-conversion. The SARS-CoV-2 sero-conversion rate, generally defined as the proportion of subjects sero-negative at baseline turning positive, can be determined.

The average duration of clinical symptoms is assessed for children and compared to that of adults. To describe the effects of underlying or previous diseases on the course of SARS-CoV-2 infection or COVID-19 disease, information from the questionnaires are used to identify risk and protective factors that can potentially influence the susceptibility and course of viral infection or COVID-19 disease. In addition, contextual factors of transmission dynamics in day care centers and indications of accompanying psychosocial burden within households are described. Due to the non-representative selection of the facilities, however, only a descriptive exploration of possible transmission factors and psychosocial burden can be made, i.e., abnormalities and patterns can be described severe.

### Sample Size Calculation

The sample size calculation is based on the effective number of cases, which is determined by a design effect due to the clustering of participants within day care centers and households. Following assumptions are considered: In general, the group size in day care centers ranges from 13 to 23 children and each group is supervised by 3 educators; households of persons who are tested positive, comprise on average 2 adults and 1.5 children. As in the emergency operation the average group size is probably smaller and under the assumption of non-participation, the sample size in each day care center should be at least about 11 children and 2–3 adults. Including the household members of those who are tested positive, at least 20 to a maximum of 30 persons per day care center should participate in the study. Purposive sampling should ensure that there are enough day care centers in which children are identified as index cases by the health authorities (aim: ~50–70% of all day care centers). This may, however, depend on the SARS-CoV-2 incidence in the population, and also on the willingness of parents to cooperate in the study. After the initial 2 months of the study all assumptions are checked and the calculation updated. Accordingly, the aim is to include 30 day care centers plus associated households with overall 450 children and 580 adults and with 35 children and 55 adults with a confirmed SARS-CoV-2 case infection (excluding the index cases).

### Data Protection and Ethics Statement

COALA is subject to strict compliance with the data protection regulations of the EU Data Protection Basic Regulation (GDPR) and the Federal Data Protection Act (BDSG). The study was planned with data protection provisions set out in the Federal Data Protection Act. The RKI-internal Commissioner for Data Protection received the COALA study concept and had no objections.

The Ethics Committee of the Berlin Medical Association has reviewed the COALA study and approved the implementation of the study (Eth-39/20). The participation in the study is voluntary and all participants are informed about the objectives and contents of the study as well as data protection and give their written informed consent; children aged from 14 to 17 years give their own written consent in addition. Every participant is assigned to a sequential study number (ANR) in order to ensure pseudonymization of the study documents and material.

COALA is registered in the German Clinical Trail Register (DRKS-ID=DRKS00023501).

## Discussion

A better and precise understanding of the factors fostering SARS-CoV-2 transmission in day care centers is essential for the development of tailored countermeasures against the spread of SARS-CoV-2 in these settings. Educational and social care in day care centers are influencing factors on the psychosocial and cognitive development of children. Prolonged closures of day care centers, induced by COVID-19, poses many challenges for families, especially for those with limited resources (30). SARS-CoV-2 infections in day care centers may spread among children and staff and, eventually, to connected households. Transmission dynamics of SARS-CoV-2 can be amplified further if infected children are not isolated, due to a general lack of clinical symptoms and insufficient testing. Understanding the dynamics of infection in the day care center setting can help adequately respond to changing conditions as the pandemic progresses, particularly with the emergence of new variants of SARS-CoV-2.

One of the strengths of COALA is the analysis of the transmission dynamics of SARS-CoV-2 in potential high-risk and non-clinical settings. The use of regular self-sampling for PCR tests over time among positive children or adults and especially their close contacts is different compared to study settings in outpatient care or hospitals. Most studies on SARS-CoV-2 take place in clinical settings, e.g., pediatric and emergency departments. There, children present with health complaints, although these are often noninfectious conditions without SARS-CoV-2-associate symptoms. These studies from clinical settings show inconsistent results on how often children are asymptomatic carriers of SARS-CoV-2 so far (31, 32). With COALA, it is possible to gather data on clinical symptoms among non-hospitalized children aged 1 to 6 years who may otherwise not have been tested for SARS-CoV-2 and diagnosed with COVID-19 at all.

### Limitations

The study is subject to some limitations. As the study design is outbreak-related, the results cannot be considered as representative for all day care center settings in Germany. COALA is a community-based study, hence data about children and adults with severe courses of diseases is potentially underrepresented, e.g., in case of hospitalization or severe clinical illness. It should also be considered that measurements take place over a time span of ~40 weeks between October 2020 and June 2021. Transmission dynamics of SARS-CoV-2 as well as contextual factors of the pandemic can potentially change over this period of time. Since self-sampling and sampling of infants is conducted in a non-clinical environment, variations in quality and yield of samples are to be expected. The quality of respiratory specimens can be diminished by the fact that self-sampling and sampling of infants is partly conducted by non-professionals. In addition to that, conditions under which the biospecimens are generally transported, e.g., temperature, can have a negative influence on the quality of the specimens.

## Conclusion

The analysis of the transmission dynamics of SARS-CoV-2 in a non-clinical setting can be a challenge for the participants and the researchers equally. Findings of the COALA study will improve the understanding of SARS-CoV-2 transmission in day care centers and corresponding households with the aim to offer better protection strategies from SARS-CoV-2 infection for day care center children and staff as well as their families. The longitudinal observation of exposed children and adults, regardless of their clinical symptoms, provides valuable insight into the infectivity and susceptibility of asymptomatic children and adults infected with SARS-CoV-2 and enables the design of accurate measures against the transmission of SARS-CoV-2 in day care centers.

## Ethics Statement

The studies involving human participants were reviewed and approved by Ethics Committee of the Berlin Medical Association (Friedrichstraße 16, 10969 Berlin, Germany). Written informed consent to participate in this study was provided by the participants' legal guardian/next of kin.

## Author Contributions

JL, ASc, SJ, AH, HP, M-LZ, AS-R, AG, JA, MS, MB, HB, JWe, EO, UB, WH, and TL contributed to the conceptualization of the study. JL, AH, HP, ASa, UK, SJ, GV, JM, and LSchr were involved in the conceptualization of biological sample procedures. SJ, ASc, AH, PR, A-KML, CC, MW, and SA were involved in the conceptualization of the interviews. JL, SJ, HP, M-LZ, ASa, JWo, BW, TK, and IH organized the recruitment strategy. AS-R and SD calculated the sample size. ASc, SJ, AH, ASa, UK, and JL prepared the first draft of the manuscript. All authors contributed to editing the first draft of the manuscript, and approved the final version.

## Funding

Corona outbreak-related examinations in day care centers—COALA is conducted by the Robert Koch Institute (RKI) and is funded by the Federal Ministry of Health under Grant Number ZMVI1-2520COR404 for the period June 2020 to December 2021. COALA is one out of four modules of the Corona day care center study: Research on the organizational, hygienic and educational challenges of emergency day care in day care centers as well as on acute respiratory diseases during the implementation of measures to contain SARS-CoV-2, which is being conducted by the German Youth Institute (DJI) together with the RKI. The Federal Ministry of Health was not involved in the design of the study or collection of data.

## Conflict of Interest

The authors declare that the research was conducted in the absence of any commercial or financial relationships that could be construed as a potential conflict of interest.

## Publisher's Note

All claims expressed in this article are solely those of the authors and do not necessarily represent those of their affiliated organizations, or those of the publisher, the editors and the reviewers. Any product that may be evaluated in this article, or claim that may be made by its manufacturer, is not guaranteed or endorsed by the publisher.
